# Targeting Sarcopenia in CKD: The Emerging Role of GLP-1 Receptor Agonists

**DOI:** 10.3390/ijms26168096

**Published:** 2025-08-21

**Authors:** Vicente Llinares-Arvelo, Carlos E. Martínez-Alberto, Ainhoa González-Luis, Manuel Macía-Heras, Orlando Siverio-Morales, Juan F. Navarro-González, Javier Donate-Correa

**Affiliations:** 1 Escuela de Enfermería Nuestra Señora de Candelaria, Carretera General del Rosario, 145, 38010 Santa Cruz de Tenerife, Spain; vllinare@ull.edu.es (V.L.-A.); extcmartina@ull.edu.es (C.E.M.-A.); 2 Doctoral and Graduate School, University of La Laguna, 38200 San Cristóbal de La Laguna, Spain; ainhoa.gonaluz@gmail.com (A.G.-L.); orlandosiverio83@gmail.com (O.S.-M.); 3 Research Unit, University Hospital Nuestra Señora de Candelaria (UHNSC), 38010 Santa Cruz de Tenerife, Spain; jnavgon@gobiernodecanarias.org; 4 Nephrology Service, University Hospital Nuestra Señora de Candelaria (UHNSC), 38010 Santa Cruz de Tenerife, Spain; mmacia25@hotmail.com; 5 GEENDIAB (Grupo Español Para el Estudio de la Nefropatía Diabética), Sociedad Española de Nefrología, 39000 Santander, Spain; 6 Instituto de Tecnologías Biomédicas, Universidad de La Laguna, 38000 Santa Cruz de Tenerife, Spain; 7 RICORS2040 (RD24/004/0022), Instituto de Salud Carlos III, 28000 Madrid, Spain

**Keywords:** GLP-1 receptor agonists, chronic kidney disease, sarcopenia, muscle mass, frailty

## Abstract

Sarcopenia is a prevalent and disabling complication of chronic kidney disease (CKD), associated with frailty, diminished quality of life, and increased morbidity and mortality. Despite its clinical significance, no pharmacological treatments are currently approved to address muscle wasting in this population. Glucagon-like peptide-1 receptor agonists (GLP-1RAs), widely used in the management of type 2 diabetes and obesity, have shown potential to support muscle mass and function through pleiotropic mechanisms. These include anti-inflammatory and antioxidant actions, improvements in insulin sensitivity and energy metabolism, and mitochondrial support. Given the high burden of sarcopenia in CKD and the frequent overlap with metabolic and cardiovascular comorbidities, GLP-1RAs may offer a novel therapeutic approach. This review examines the biological plausibility and emerging evidence supporting the role of GLP-1RAs in preserving muscle health in CKD, highlighting the need for targeted clinical trials and mechanistic investigations to establish their efficacy in this high-risk group.

## 1. Introduction

Sarcopenia is defined as a progressive musculoskeletal disease characterized by the loss of muscle mass, strength, and/or physical performance [1]. Although traditionally associated with aging, it is increasingly recognized in younger populations with chronic diseases—particularly those with metabolic and inflammatory components, such as chronic kidney disease (CKD). Obesity, which often coexists with aging, can further exacerbate age-related declines in muscle mass and function through mechanisms including chronic low-grade inflammation, insulin resistance, and altered muscle metabolism [2], thereby compounding the risk of sarcopenia. Patients with chronic kidney disease (CKD) exhibit an accelerated aging phenotype, leading to early declines in physical function and nutritional status, including marked reductions in lean body and skeletal muscle mass [3,4,5,6,7,8,9,10]. These changes contribute to a heightened risk of sarcopenia in renal patients, especially in those with end-stage renal disease.

Today, both CKD and sarcopenia are recognized as major public health concerns. CKD affects approximately 9–13% of the global population [11], and epidemiological models suggest that the incidence of sarcopenia among older adults could reach 72.4% by 2045 [12], a projection that highlights the urgent need for targeted interventions in these high-risk groups. In fact, sarcopenia is highly prevalent across all stages of CKD. A 2024 meta-analysis including over 42,000 patients estimated the global prevalence at 25%, with 43% of individuals exhibiting low muscle strength [7]. In this study, the prevalence of severe sarcopenia was significantly higher in dialysis patients compared to non-dialysis counterparts (26% vs. 3%) [7]. This striking burden reflects the convergence of multiple CKD-related pathophysiological drivers that include systemic inflammation, insulin resistance, uremic toxin accumulation, metabolic acidosis, hormonal imbalances, and protein–energy wasting [3,8].

The consequences of sarcopenia in CKD are profound, being independently associated with a wide spectrum of adverse outcomes, including physical disability, frailty, reduced quality of life, depression, postoperative complications, and graft failure in kidney transplant recipients. Moreover, it is a well-established predictor of increased hospitalization and all-cause and cardiovascular mortality [1,4,9,10,13]. Consequently, the management of sarcopenia is of critical importance in improving patient outcomes in CKD. Although exercise and nutritional interventions remain foundational for the management of sarcopenia, no pharmacological therapies have consistently demonstrated efficacy—either in CKD-specific contexts or in broader clinical populations—and the clinical implementation of existing strategies remains suboptimal [14].

In recent years, significant therapeutic advances have emerged for individuals with CKD, particularly in the area of cardiometabolic protection. Among these, glucagon-like peptide-1 receptor agonists (GLP-1RAs)—originally developed for glycemic control in type 2 diabetes mellitus (T2DM)—have gained relevance. GLP-1RAs have demonstrated robust cardiovascular and renal benefits, including reductions in major adverse cardiovascular events (MACE), new-onset macroalbuminuria, and estimated glomerular filtration rate (eGFR) decline [15,16,17,18,19]. As a consequence of these studies, GLP-1RAs are now recommended for patients with T2DM and comorbid CKD. GLP-1RAs act by stimulating glucose-dependent insulin secretion, suppressing glucagon release, and enhancing pancreatic β-cell function [20,21,22]. In addition, they induce weight loss by slowing gastric emptying and reducing appetite [23,24]. Due to their weight-reducing effects, certain GLP-1RAs—such as liraglutide (Saxenda®) and semaglutide (Wegovy®)—have been approved for obesity treatment. More recently, tirzepatide (marketed as Mounjaro® and Zepbound®), a dual glucose-dependent insulinotropic polypeptide (GIP) and GLP-1 receptor agonist, has been also approved for chronic weight management.

However, the rapid and substantial weight loss induced by these agents has raised concerns about potential muscle mass and strength loss—especially in populations at a heightened risk of sarcopenia, such as renal patients [25]. Despite these concerns, emerging evidence suggests that GLP-1RAs may positively influence skeletal muscle metabolism and function—an area of particular interest in sarcopenia prevention and treatment. These potential benefits are attributed to their pleiotropic effects, including anti-inflammatory activity, improved metabolic efficiency, and possible direct anabolic or anti-catabolic effects on skeletal muscle tissue. Together, these features position GLP-1RAs as promising candidates for the prevention and management of sarcopenia in CKD and other high-risk populations [26,27,28,29,30].

## 2. Risk Factors and Mechanisms Contributing to Sarcopenia in CKD

**Figure 1:**
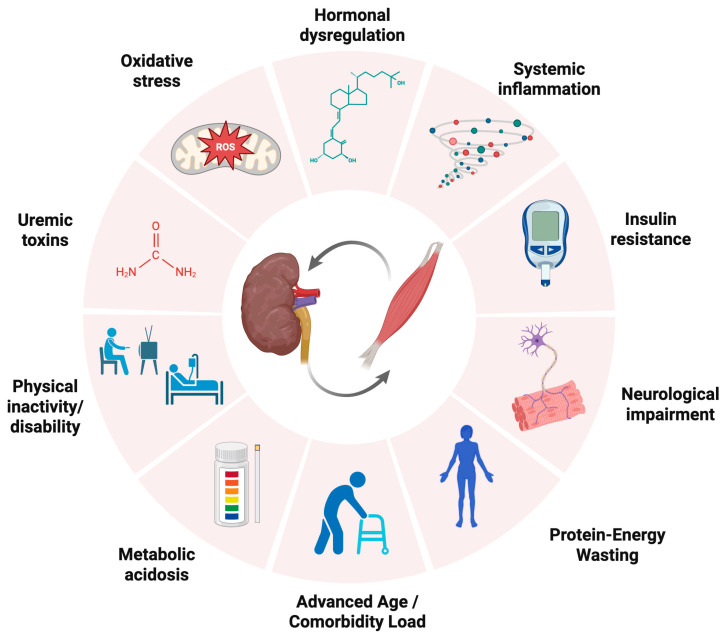
Risk factors for the progression of sarcopenia in patients with CKD.

Sarcopenia in CKD results from a complex interplay among systemic and disease-specific mechanisms that impair muscle protein homeostasis, regeneration, and function (Figure 1). Similarly to the general population, aging and decreased physical activity constitute important risk factors for sarcopenia, with increased prevalence in aging CKD patients [31]. Together with aging, specific CKD-related risk factors for the progression of sarcopenia include chronic systemic inflammation, oxidative stress, uremic toxin accumulation, metabolic acidosis, and hormonal dysregulation [32,33].

A key signaling node in this context is the mammalian target of rapamycin (mTOR), a central anabolic regulator of muscle metabolism. Under normal conditions, the activation of the insulin/phosphoinositide 3-kinase/protein kinase B (PI3K/Akt)/mTOR signaling pathway by growth factors (e.g., insulin-like growth factor 1 (IGF-1), insulin), amino acids, and mechanical stimuli promotes protein synthesis, suppresses autophagy, and maintains muscle mass. In CKD, multiple pathological processes converge to suppress this pathway, thereby reducing protein synthesis, enhancing autophagy inhibition, and facilitating proteolysis via the ubiquitin–proteasome system (UPS).

Inflammation plays a central role in the initiation and progression of sarcopenia through catabolic signaling pathways and muscle degradation. Patients with CKD present a persistent systemic low-grade inflammatory status that has been independently associated with sarcopenia. It is not only reflected by high malnutrition–inflammation scores [33,34,35] but also by high levels of inflammatory markers, including high-sensitive C-reactive protein (hs-CRP) [32,36,37,38], ß2-microglobulin [32,39], and interleukin (IL)-4 and -6 [32,36,38,39]. Many of these actions are mediated by the activation of the nuclear factor kappa-light-chain-enhancer of activated B cells (NF-κB) signaling pathway, which is considered a central mediator of inflammation-induced muscle catabolism. Upon cytokine stimulation (e.g., TNF-α), NF-κB is released from its inhibitor IκB, translocates to the nucleus, and upregulates the transcription of E3 ubiquitin ligases *Atrogin-1* and *Muscle RING-finger protein-1* (*MuRF-1*) while suppressing anabolic IGF-1 signaling, indirectly suppressing mTORC1 and accelerating UPS-mediated proteolysis [40,41]. IL-6 also disrupts PI3K/Akt signaling, further impairing mTORC1-dependent protein synthesis [42,43].

Additionally, oxidative stress, driven by excess reactive oxygen species (ROS) and mitochondrial dysfunction, directly inhibits Akt/mTORC1 signaling at multiple levels—both at or upstream of Akt (e.g., affecting PI3K) and downstream of Akt (e.g., directly impairing mTOR or translation machinery) [44,45,46]. This not only suppresses protein synthesis but also triggers ALP activation through mTORC1 inhibition and the activation of stress-responsive transcription factors (forkhead box O [FoxO], p38 MAPK), which increases autophagy gene expression (microtubule-associated protein 1 light chain 3 [LC3], BCL2 interacting protein [BNIP3], Beclin-1) [47]. This dual action both relieves mTOR-dependent autophagy repression and increases the expression of autophagy machinery, culminating in increased protein degradation and muscle atrophy. Moreover, as CKD progresses, excessive oxidative stress occurs with increased ROS production, thereby promoting the synthesis and release of pro-inflammatory cytokines [48], together with the consequent inflammation-mediated muscular atrophy, further exacerbating the extent of atrophy.

Metabolic acidosis can further exacerbate protein catabolism in CKD by activating the UPS and caspase-3 pathways [49], promoting insulin and growth hormone (GH) resistance [50]. Furthermore, metabolic acidosis stimulates the ALP in skeletal muscle via the inhibition of mTORC1 and activation of FoxO [51]. Finally, acidosis also contributes to inflammation and mitochondrial dysfunction, contributing to the impairment of anabolic signaling. Clinically, correcting acidosis with oral bicarbonate not only slows CKD progression but also improves muscle mass, strength, and nutritional status in patients [52,53,54].

Patients with CKD also experience hormonal imbalances, including reduced levels of anabolic IGF-1 and increased levels of myostatin, further contributing to the catabolic state. As mentioned above, IGF-1 promotes muscle health by activating the PI3K/Akt/mTOR pathway, stimulating protein synthesis, and inhibiting protein degradation [55]. The reduction in IGF-1 levels disrupts this activation, impairing muscle protein synthesis and increasing protein degradation [56]. The rise in myostatin levels in CKD is induced by inflammatory cytokines, reinforcing the link between inflammation and muscle atrophy [57]. Elevated levels of myostatin contribute significantly to muscle atrophy by inhibiting muscle protein synthesis through the suppression of the Akt/mTOR pathway and enhancing protein degradation via the activation of the UPS [58,59]. It also impairs the regenerative capacity of muscle by suppressing satellite cell activation and promotes muscle fibrosis and fat infiltration [43].

Insulin resistance also contributes to muscle loss by disrupting glucose uptake, therefore blunting the anabolic effects in skeletal muscle derived from the activation of the PI3K/Akt/mTOR pathway. Additionally, it enhances protein degradation via the FoxO pathway, contributing to muscle wasting [60].

The accumulation of uremic toxins, such as indoxyl sulfate and p-cresyl sulfate, also contributes to muscle atrophy in CKD by impairing muscle mitochondrial function and IGF-1 signaling [61,62,63,64]. Meanwhile, high levels of uric acid and advanced glycation end products, thyroid hormone imbalances, and vitamin D deficiency contribute to muscle dysfunction and are associated with sarcopenia in CKD patients.

Patients with CKD frequently develop protein–energy wasting (PEW), a multifaceted malnutrition syndrome driven by anorexia, malabsorption, and nutrient loss during dialysis, all of which exacerbate muscle degradation [65,66]. Impaired appetite in CKD often results from uremic toxins, gastrointestinal disturbances, and chronic inflammation, leading to anorexia and reduced food intake—factors strongly associated with increased PEW and mortality risks [67]. Additionally, inflammation-induced gastrointestinal dysfunction contributes to malabsorption, further limiting nutrient availability [68]. Dialysis procedures—both hemodialysis and peritoneal—lead to significant protein and amino acid losses, intensify resting energy expenditure, and promote a negative nitrogen balance, directly worsening muscle catabolism [68].

Finally, neurological dysfunction in CKD, including uremic peripheral neuropathy, occurs in approximately 60–100% of dialysis patients and leads to impaired neuromuscular activation, reduced motor coordination, and muscle weakness—key contributors to muscle atrophy in this population [69,70]. The concurrent cognitive impairment in CKD, driven by the accumulation of neurotoxic uremic metabolites and chronic inflammation, further diminishes physical activity and disrupts central brain–muscle communication, compounding the risk of sarcopenia. Finally, physical inactivity, also resulting from fatigue, anemia, comorbidities, and limited mobility, leads to reduced mechanical stimulus essential for muscle maintenance [71].

## 3. Evidence from Clinical Studies

There are very few studies directly assessing body composition outcomes specifically in CKD patients treated with GLP-1RAs or related therapies. Most large trials [like SUSTAIN (Semaglutide Unabated Sustainability in Treatment of Type 2 Diabetes) and SURPASS (Study of Tirzepatide in Participants with Type 2 Diabetes Under Various Regimens to Assess Efficacy and Safety)] either exclude advanced CKD patients or do not report detailed body composition data stratified by CKD status. However, several randomized clinical trials (RCTs) and mechanistic studies offer indirect but compelling evidence of their potential to support muscle preservation alongside metabolic improvements.

### 3.1. Evidence from Clinical Studies in CKD or CKD-Stratified Populations

Although most major GLP-1RA trials have excluded patients with advanced CKD, some recent studies have included CKD populations or conducted subgroup analyses. Therefore, large clinical trials have demonstrated that GLP-1RAs, such as liraglutide, semaglutide, and dulaglutide, reduce albuminuria, slow the progression of kidney disease, and improve cardiovascular outcomes in patients with CKD (Table 1) [15,16,17,18,19]. The results of these trials show improvements in renal and metabolic parameters that could be potentially relevant to sarcopenia. Slower CKD progression may reduce exposure to uremic toxins, which—as mentioned above—are known to impair mitochondrial function, increase oxidative stress, and inhibit muscle regeneration. Similarly, better glycemic control and lower insulin resistance may also reduce muscle protein breakdown and improve the anabolic response to nutrients and insulin. Moreover, reductions in systemic inflammation—suggested by the surrogate marker albuminuria—may attenuate the catabolic signaling associated with inflammation.

In the REWIND (Researching Cardiovascular Events with Weekly Incretin in Diabetes) trial, weekly dulaglutide at 1.5 mg reduced the risk of a composite kidney outcome—including new macroalbuminuria, ≥30% eGFR decline, or need for dialysis—by 15% over 5.4 years, primarily due to a 23% reduction in macroalbuminuria [17]. Similarly, the LEADER (Liraglutide Effect and Action in Diabetes: Evaluation of Cardiovascular Outcome Results) and SUSTAIN-6 trials both demonstrated significant renal benefits of GLP-1RAs in patients with T2DM at high cardiovascular risk. In LEADER, daily liraglutide at 1.8 mg led to a 22% reduction in a composite renal endpoint (new macroalbuminuria, doubling of creatinine, renal replacement therapy, or renal death), largely driven by a 26% drop in macroalbuminuria [15]. In SUSTAIN-6, semaglutide weekly (0.5–1.0 mg) decreased renal events by 36%, with a striking 46% reduction in new-onset macroalbuminuria [16].

Most notably, the FLOW (Evaluate Renal Function with Semaglutide Once Weekly) trial—dedicated specifically to renal outcomes—found that semaglutide at 1 mg weekly reduced the risk of major kidney events—including kidney failure, sustained ≥50% decline in eGFR, or death from kidney or cardiovascular causes—by approximately 24% compared to a placebo [19]. Semaglutide also slowed the annual decline in kidney function by about 1.16 mL/min/1.73 m^2^ and showed consistent benefits across subgroups, regardless of baseline eGFR, albuminuria or concurrent SGLT2 inhibitor use.

Additionally, the AMPLITUDE-O (Effect of Efpeglenatide on Cardiovascular Outcomes) trial evaluated efpeglenatide in patients with T2DM at high cardiovascular risk, including a significant subset with CKD (eGFR between 25 and 59.9 mL/min/1.73 m^2^) [18]. Over a median follow-up period of 1.8 years, efpeglenatide significantly reduced the risk of major adverse cardiovascular events (MACE) by 27% and, importantly, showed a 32% reduction in a composite renal endpoint (kidney function decline or macroalbuminuria) (HR 0.68; 95% CI 0.57–0.79; *p* < 0.001).

Of particular note, the AWARD-7 trial was among the few RCTs specifically designed to compare GLP-1RA treatment in patients with moderate-to-severe CKD (stages 3–4). This study found that dulaglutide preserved kidney function more effectively than insulin glargine over 52 weeks, with a significantly slower decline in eGFR, especially in those with baseline macroalbuminuria [72].

Together, these large trials demonstrate that GLP-1RAs—across both human-analog and exendin-based agents—provide meaningful kidney protection in patients with T2DM, especially through reducing albuminuria, with semaglutide and efpeglenatide also showing broader benefits against eGFR decline and hard kidney events.

Preliminary body composition data in CKD populations also arise from smaller ancillary studies. Thus, a small study including only 21 patients with T2DM on hemodialysis that received either dulaglutide (*n* = 11) or teneligliptin (*n* = 10) over six months showed significant reductions in both fat mass and skeletal muscle mass in those treated with GLP-1RAs [73]. While these results raise concerns about muscle loss in dialysis patients, the small cohort size and lack of functional outcome measures highlight the need for larger, well-controlled trials before drawing firm conclusions.

### 3.2. Findings from Clinical Trials in the General T2DM Population: Muscle-Related Outcomes

Several large-scale RCTs have evaluated GLP-1RAs, primarily focusing on glycemic control and cardiovascular protection. Although these trials were not explicitly designed to evaluate skeletal muscle strength or sarcopenia, accumulating evidence suggests that GLP-1RAs may exert neutral to potentially beneficial effects on muscle health (Table 2).

Some RCTs have included determinations of physical function and muscle performance. Thus, in patients with advanced heart failure or T2DM, liraglutide showed no significant difference compared to a placebo in measures of endurance, including maximal oxygen uptake (VO_2_ max), cycle ergometry duration, and 6-min walk distance. These findings suggest the preservation of physical performance capacity without detrimental effects on muscle function [80]. Similarly, in individuals with obesity—with or without T2DM—liraglutide was associated with improvements in self-reported physical function [81].

Only a few studies have included accurate measurements of body composition with weight loss endpoints, including absolute and relative losses of lean mass, typically assessed by dual-energy X-ray absorptiometry (DXA). This was the case for SUSTAIN-8, which compared the administration of semaglutide 1.0 mg to that of canagliflozin daily [82]. Semaglutide demonstrated greater total weight loss, and, importantly, a DXA-based substudy of the SUSTAIN-8 trial revealed that, despite the absolute decline, both groups experienced an increase in the relative proportion of LM to total body weight (semaglutide: +1.2%; canagliflozin: +1.1%), suggesting a favorable effect on body composition and potential mitigation of sarcopenic risk [27].

Similarly, a post hoc analysis of the STEP 1 (Semaglutide Treatment Effect in People with Obesity) trial, with adults with obesity or overweight without diabetes receiving semaglutide 2.4 mg for 68 weeks, also included data from DEXA scans [26,83]. The results showed that, although the absolute LM decreased along with fat mass, the relative proportion of lean mass increased, highlighting preferential adipose tissue targeting by semaglutide—a desirable feature for sarcopenia-risk populations [26]. In detail, total LM decreased 9.7%, but the proportion of LM relative to total body mass increased by 3.0%. Similar results were obtained in a SURMOUNT-1 DXA substudy (a study of tirzepatide—a dual glucose-dependent insulinotropic polypeptide (GIP)—and GLP-1RAs in participants with obesity or overweight) that demonstrated a large decrease in fat mass (33.9%) and moderate lean mass loss (10.9%) compared to a placebo (2.6%) after 72 weeks [74].

Notably, the SURPASS clinical trial program, which evaluated the efficacy and safety of tirzepatide, also provided valuable data on body composition. In the SURPASS-3 and SURPASS-5 substudies—both of which included DXA assessments—tirzepatide led to significant reductions in visceral and subcutaneous adipose tissue, accompanied by improvements in metabolic parameters, while preserving a greater proportion of lean mass [75,76]. This proportionate preservation is especially relevant in the context of CKD, where weight loss therapies often risk exacerbating muscle wasting.

Moreover, a recent post hoc analysis of the SURPASS-3 trial included magnetic resonance imaging (MRI)-based assessments to evaluate changes in muscle and fat within the thigh [77,78]. After 52 weeks, patients treated with tirzepatide demonstrated greater reductions in visceral and hepatic fat compared to those on insulin degludec, despite similar or greater weight loss overall [77]. Moreover, tirzepatide treatment was associated with thigh muscle volume changes that were roughly proportional to the overall weight loss, indicating no disproportionate muscle atrophy [78]. Importantly, tirzepatide also significantly reduced intramuscular fat infiltration (muscle fat content) in the thigh, surpassing reductions expected purely from weight loss alone—suggesting an improvement in muscle quality.

Similarly, two complementary trials also employed MRI to quantify changes in adipose and muscle compartments in response to liraglutide in individuals with overweight or obesity and an elevated cardiovascular risk [84,85]. Compared with a placebo, liraglutide significantly reduced visceral adipose tissue (VAT) by 12.5%, hepatic fat content by 31.5%, and epicardial fat by 13.4%. Notably, these reductions were disproportionately greater than expected from weight loss alone, suggesting direct pharmacologic effects of liraglutide on ectopic fat depots. A subsequent post hoc analysis of this cohort aimed to evaluate the thigh muscle composition, including muscle fat infiltration and muscle volume, and revealed a significant 2.87% absolute reduction in intramuscular fat infiltration, while changes in muscle volume were modest and not statistically significant after body size adjustment. Moreover, the proportion of participants with an adverse muscle composition—defined as high fat infiltration with low muscle mass—declined from 11.0% to 8.2% in the liraglutide group, with no such improvement observed in the placebo arm [86]. Collectively, these studies provide robust, imaging-based evidence that liraglutide preferentially reduces ectopic and visceral fat while concurrently enhancing skeletal muscle quality. These findings align with meta-analyses showing that GLP-1RAs and GLP-1/GIP co-agonists reduce lean mass by approximately 25% of total weight loss, which is consistent with the expected lean mass loss from weight reduction, rather than disproportional muscle loss [86].

Some other RCTs also apport indirect data about the muscle health implications of the administration of GLP-1RAs. The previously mentioned LEADER and SUSTAIN-6 trials, conducted in patients with T2DM at high cardiovascular risk, demonstrated a significant decrease in major adverse cardiovascular events after semaglutide or liraglutide treatment, respectively [15,16]. While muscle-specific parameters were not directly evaluated, the systemic anti-inflammatory and metabolic improvements observed may contribute to the preservation of muscle mass and function in this population, especially in frail subgroups like patients with CKD. Similarly, the AWARD-11 trial tested higher dulaglutide doses in T2DM patients, reporting significant body weight reductions with no excessive loss of lean mass. Although muscle-specific measures were absent, the overall favorable body composition outcomes imply potential muscle preservation [79]. Finally, efpeglenatide, a long-acting GLP-1RA, showed improved cardiovascular and renal outcomes in high-risk diabetic populations in the previously mentioned AMPLITUDE-O trial [18]. Again, while direct muscle assessments were lacking, the potent anti-inflammatory, anti-catabolic, and endothelial effects may indirectly benefit skeletal muscle, especially in comorbid states predisposing patients to sarcopenia.

Beyond imaging techniques, additional studies and exploratory analyses in populations with T2DM further support the preferential fat loss effects of GLP-1RAs through assessments of body composition through bioelectrical impedance analysis (BIA). Thus, an observational study in adults initiating oral semaglutide reported a total weight loss of 4.0 kg, including an average reduction of 3.2 kg in fat mass, with no significant change in skeletal muscle mass after 16 weeks [87]. Similarly, a pilot, prospective observational study involving elderly adults with obesity found that semaglutide, combined with caloric restriction and exercise interventions for 3 months, preserved appendicular LM while reducing the total fat mass, leading to improved physical function scores [88]. Likewise, a recent real-world quasi-experimental study evaluated the effects of semaglutide over 24 weeks. Using BIA assessments, the authors found that weight loss—approximately 9.5% of the baseline body weight—was primarily driven by fat mass reduction, while skeletal muscle mass and fat-free mass were preserved. Notably, the phase angle remained unchanged, suggesting that semaglutide-induced weight loss did not compromise muscle quality or cellular health [89]. Finally, improvements in health-related quality of life (HRQoL) and functional performance tests (such as the six-minute walk test) have been reported with GLP-1RA therapy in some cohorts, although data remain limited and are primarily derived from observational studies [90].

Although these landmark GLP-1RA trials were not specifically designed to assess sarcopenia, their consistent demonstration of fat mass reduction with relative lean mass preservation, combined with improvements in systemic metabolic and inflammatory profiles, strongly supports the hypothesis that GLP-1RAs may help to prevent or slow muscle wasting. These secondary insights underscore the rationale for dedicated studies targeting sarcopenic populations.

## 4. Evidence from Preclinical Studies

Diverse preclinical studies collectively highlight the multifaceted actions of GLP-1RAs on skeletal muscle biology. In general terms, this experimental research points to the existence of direct and indirect beneficial effects on muscle remodeling and function. Although most experimental work has been conducted in general metabolic models, the findings are highly relevant to CKD, a condition characterized by systemic inflammation, oxidative stress, mitochondrial dysfunction, and insulin resistance—all key contributors to muscle wasting.

### 4.1. In Vitro Studies

In vitro studies using murine myogenic cell lines (particularly C2C12 myoblasts and myotubes) have consistently shown that GLP-1RAs exert cytoprotective, anabolic, and metabolic benefits that could counteract the drivers of muscle atrophy in CKD. Thus, liraglutide reduces cellular senescence and enhances myogenic differentiation under diabetic-mimicking conditions (high glucose and palmitate) via modulation of the mechanosensitive YAP (Yes-associated protein) and TAZ (transcriptional coactivator with PDZ-binding motif) signaling pathways [90,91]. These pathways are also altered in CKD, where uremic toxins and insulin resistance impair muscle regeneration.

Similarly, exendin-4 improves mitochondrial respiration, upregulates oxidative fiber-type gene expression, and enhances glucose uptake through AMPK activation—a central node in energy regulation that is disrupted in CKD-related muscle dysfunction [92,93]. These benefits are nullified by AMPK knockdown, highlighting the pivotal role of GLP-1R/AMPK signaling. Furthermore, exendin-4 upregulates thermogenic and fatty acid oxidation genes such as uncoupling protein 1 (UCP1), peroxisome proliferator-activated receptor alpha (PPARα), and β3-adrenergic receptor, via a PKA-dependent mechanism, suggesting increased metabolic flexibility, which may mitigate lipid-induced toxicity commonly seen in uremic muscle.

In inflammatory conditions, the GLP-1RA PF1801 protects C2C12 myotubes from FAS ligand (FasL)-induced necroptosis by activating AMPK, promoting the degradation of PGAM5, increasing antioxidant gene expression, and reducing ROS accumulation [94]—mechanisms that are particularly relevant in the oxidative milieu of CKD.

GLP-1RAs also demonstrate efficacy against catabolic stimuli. Exendin-4 attenuates dexamethasone-induced muscle atrophy via the activation of the PI3K/Akt/mTOR pathway, downregulation of atrogenes *MuRF-1* and *Atrogin-1*, and restoration of glucose transporter type 4 (GLUT4) expression [28,30,95]. These effects translate into enhanced protein synthesis and improved insulin-independent glucose uptake in both L6 rat skeletal muscle cells and C2C12 myotubes, again via AMPK signaling [96,97].

Additionally, GLP-1RAs improve autophagic flux in myotubes, through Sestrin2 (SESN2)- and LC3B-dependent mechanisms, supporting cellular stress resilience and protein turnover—key processes disrupted in CKD-induced muscle wasting [98].

Beyond direct effects on muscle cells, GLP-1RAs may also enhance vascular support, a critical component of muscle maintenance in CKD, where microvascular dysfunction is common. Exendin-4 promotes the redifferentiation of vascular smooth muscle cells via AMPK/SIRT1/FOXO3a signaling [99] and induces vasodilation through cAMP/PKA activation and RhoA/ROCK inhibition [99,100,101], potentially improving oxygen and nutrient delivery to skeletal muscle.

However, emerging evidence also suggests that chronic GLP-1 exposure may carry context-dependent drawbacks. A recent study by Huang et al. (2024) reported that prolonged GLP-1 treatment in C2C12 myoblasts led to impaired differentiation, defective GLUT4 translocation, and reduced mitochondrial ATP production [102]. These findings raise concerns about the long-term impact of sustained GLP-1 signaling on muscle regeneration, especially in aging or uremic muscle, where the regenerative capacity is already compromised.

In summary, in vitro evidence highlights the multifactorial actions of GLP-1RAs on skeletal muscle cells—including anti-senescent, anti-necroptotic, anabolic, and metabolic effects—primarily mediated through the AMPK, PKA, PI3K/Akt/mTOR, and mitochondrial signaling pathways. Indirect vascular benefits further suggest that GLP-1RAs may support systemic muscle health, especially under metabolic stress or age-related decline.

### 4.2. Animal Model Studies

In vivo studies extend and reinforce in vitro findings, demonstrating that GLP-1RAs exert multisystem benefits on muscle mass, structure, and function in models that share pathophysiological features with CKD—including oxidative stress, inflammation, metabolic dysregulation, and mitochondrial dysfunction.

In aged mouse models, exendin-4 has been shown to preserve skeletal muscle mass and improve function by activating the AMPK–SIRT1–PGC-1α axis, a key regulator of mitochondrial biogenesis and cellular energy homeostasis. These molecular effects result in reduced oxidative stress and the preservation of muscle fiber integrity, which translate into improved grip strength and endurance capacity [93].

In models of diet-induced sarcopenic obesity, semaglutide not only reduces adiposity and systemic inflammation—both of which are prevalent in CKD—but also enhances relative skeletal muscle mass and histological integrity. Improvements include increased an fiber cross-sectional area, density, sarcomere length, and mitochondrial content. Metabolomic profiling further indicates favorable shifts in amino acid, lipid, and organic acid pathways, suggesting enhanced anabolic signaling and metabolic efficiency [103], which may help to counteract uremic metabolic inflexibility.

Similarly, in liver disease-induced sarcopenia (diabetic KK-Ay mice fed a 3,5-diethoxycarbonyl-1,4-dihydrocollidine [DDC] diet), semaglutide preserved the muscle fiber architecture, suppressed catabolic gene expression, and reduced inflammation and oxidative stress—ultimately improving muscle strength and physical performance [30].

In diabetic *db*/*db* and spontaneously diabetic torii (SDT) fatty rats, liraglutide and dulaglutide restored the fiber size, enhanced grip strength, maintained mitochondrial enzyme activity (citrate synthase, cytochrome c oxidase), inhibited the expression of *Atrogin-1* and *MuRF-1*, suppressed necroptosis, and promoted myogenesis via OPA–TLR9 signaling [101,104,105,106]. Moreover, GLP-1 overexpression or exendin-4 increased endurance, oxidative fiber proportions, glycogen storage, and muscle glucose uptake through AMPK-mediated metabolic rewiring [93].

Furthermore, in a hindlimb unloading model—used to simulate disuse-related sarcopenia, a mechanism overlapping with the inactivity seen in advanced CKD—GLP-1RA administration attenuated muscle loss, preserved mitochondrial content, and reduced oxidative stress markers [107]. These results are especially relevant to immobility-related muscle wasting in dialysis-dependent and frail CKD patients.

Notably, combining semaglutide with activin receptor II blockade preserves lean mass under caloric restriction, offering a potential synergy in CKD, where weight loss and muscle wasting often co-occur [108]. These findings underscore that GLP-1RAs—through mitochondrial enhancement, anti-inflammatory/anti-catabolic signaling, myogenic stimulation, and synergy with anabolic therapies—are promising candidates for muscle-preserving strategies in CKD-associated sarcopenia.

## 5. Potential Mechanisms of GLP-1RAs in Sarcopenia in CKD

The potential benefits of GLP-1RAs in preventing or mitigating sarcopenia in patients with CKD are gaining increasing attention. However, the precise mechanisms underlying these effects—particularly whether they partly result from direct action on muscle cells—remain incompletely understood. Thus, GLP-1R mRNA has been identified in several human tissues, including the pancreas, lung, kidney, hypothalamus, stomach, and heart, but not consistently in liver or adipose tissue [109]. The expression of GLP-1R in human skeletal muscle remains controversial. While some animal studies have reported GLP-1R expression in myocytes, human data are scarce and inconsistent, raising questions about the functional relevance of classical GLP-1 signaling within muscle tissue [109,110,111].

Notably, although limited, protein-level evidence supports the presence of GLP-1R in skeletal muscle. Western blot analyses using lysates from differentiated human muscle satellite cells and skeletal muscle biopsies have detected a ~53 kDa band consistent with GLP-1R, with expression levels modulated by the extracellular glucose concentration during myogenic differentiation [112]. This suggests that GLP-1R expression in muscle may be metabolically regulated. In rodents, GLP-1R protein has been confirmed in the tibialis anterior muscle of wild-type but not Glp1r-knockout mice, validating antibody specificity [28]. Furthermore, GLP-1RA treatment in these models activated downstream PKA and Akt signaling and reduced the expression of atrophy-related proteins such as myostatin, MuRF-1, and Atrogin-1, indicating that muscle GLP-1Rs are not only present but functionally active.

Despite these uncertainties, accumulating evidence from preclinical models supports the existence of both direct and indirect mechanisms by which GLP-1RAs may influence skeletal muscle homeostasis. In the context of CKD—where systemic inflammation, oxidative stress, insulin resistance, and catabolic signaling contribute to muscle wasting—GLP-1RAs may exert protective effects through the modulation of metabolic, inflammatory, vascular, and neuroendocrine pathways (Table 3).

In CKD, insulin resistance, chronic inflammation, oxidative stress, and mitochondrial dysfunction contribute to muscle wasting. Notably, GLP-1RAs enhance insulin secretion and action, which may support muscle anabolism through the activation of the PI3K/Akt/mTOR pathway, leading to increased protein synthesis and reduced proteolysis [113]. Additionally, GLP-1RAs promote GLUT4 translocation and improve glucose uptake in skeletal muscle, potentially through this indirect modulation of insulin sensitivity and by reduced glucotoxicity [96,113,114,115]. By improving glycemic control and reducing glucotoxicity, GLP-1RAs may restore PI3K/Akt/mTOR signaling and suppress catabolic FoxO-driven transcription, thereby alleviating proteolytic stress and supporting muscle integrity [116,117,118].

Their anti-inflammatory properties—observed across tissues including kidney and skeletal muscle—are mediated by the suppression of NF-κB and NLRP3 inflammasome activity and the modulation of macrophage polarization [119,120,121,122,123,124,125,126,127,128,129,130]. In preclinical polymyositis models and aged mice, GLP-1RA treatment reduced inflammatory cytokines and muscle atrophy markers *MuRF-1* and *Atrogin-1* [94,95].

Oxidative stress is a key driver of muscle degradation in CKD. GLP-1RAs attenuate ROS production through NADPH oxidase inhibition and the upregulation of antioxidant enzymes like SOD and GPx [130,131,132]. Additionally, they enhance mitochondrial biogenesis via AMPK–SIRT1–PGC-1α signaling, improving ATP production and reducing mitochondrial-derived ROS in skeletal muscle [133,134,135,136,137].

Furthermore, although direct data are limited, GLP-1RAs may modulate autophagy and apoptosis in skeletal muscle under uremic or hyperglycemic stress. In other tissues, GLP-1RAs restore autophagic flux and prevent ER stress-induced apoptosis via AMPK activation and mTOR modulation [138,139,140,141,142]. These mechanisms could similarly preserve myocyte viability in CKD-related sarcopenia.

The preservation of neuromuscular junction (NMJ) integrity—another contributor to muscle function—is supported by the neuroprotective actions of GLP-1RAs in CNS and ALS models through PI3K/Akt and cAMP/PKA/CREB signaling [143,144,145,146]. Although speculative in the CKD context, these findings hint at potential benefits for motor unit maintenance.

Lastly, vascular dysfunction is common in CKD and contributes to impaired muscle perfusion. GLP-1RAs exert endothelial-protective effects by enhancing nitric oxide bioavailability, reducing oxidative stress, and promoting capillary recruitment—all of which may improve muscle oxygenation and nutrient delivery [147,148,149,150].

Together, these diverse actions—spanning metabolic, inflammatory, oxidative, mitochondrial, and vascular domains—underscore the therapeutic potential of GLP-1RAs in counteracting sarcopenia in CKD, even in the absence of confirmed GLP-1R expression in human muscle cells.

## 6. Conclusions and Future Perspectives

The dramatic increase in the use of incretin-mimetic drugs, particularly GLP-1RAs, to treat obesity, has rapidly outpaced the development of evidence-based guidelines addressing their broader clinical implications. Among emerging concerns is their potential effects on skeletal muscle health—especially in individuals at high risk for sarcopenia, such as patients with CKD. Although GLP-1RAs have demonstrated the ability to reduce fat mass and preserve lean mass in some populations with T2DM, their specific impact on muscle mass and function in CKD remains unclear. Sarcopenia-related outcomes such as muscle strength, gait speed, and composition measurements using validated tools like DXA or BIA methods are rarely used as primary endpoints in clinical trials assessing GLP-1RAs.

Despite compelling biological plausibility, the current evidence is insufficient to determine whether GLP-1RAs can prevent or reverse muscle loss associated with weight loss therapies. Moderate exercise has been shown to support healthier weight loss and lean mass preservation when combined with GLP-1RAs [151,152], reinforcing the idea that combined interventions may be necessary to optimize outcomes. However, to date, there are no clinical trials that have assessed the effects of GLP-1RAs on skeletal muscle mass, strength, or function in people with sarcopenia.

Future research must address this gap through large-scale RCTs that incorporate validated diagnostic criteria such as those defined by the European Working Group on Sarcopenia in Older People (EWGSOP2), the Asian Working Group for Sarcopenia (AWGS), or the Sarcopenia Definition and Outcomes Consortium [153,154,155]. These trials should prioritize accurate body composition assessments, preferably using DXA, which offers a favorable balance of precision, safety, and cost. While indirect tools such as BIA and air displacement plethysmography may offer quick and non-invasive alternatives, they are generally less reliable and more susceptible to variability from hydration and operator error.

Moreover, sarcopenia is now defined not only by reduced muscle mass but also by impaired strength and physical performance. Improving muscle functionality—including strength, endurance, and mobility—remains critical in maintaining independence and reducing morbidity. Therefore, future studies should evaluate interventions that preserve or enhance both muscle mass and function, considering sarcopenia’s multifactorial nature.

GLP-1RAs may offer particular advantages in several high-risk groups: older adults with obesity who require fat loss with muscle preservation; patients with T2DM, in whom sarcopenic obesity and frailty are common; individuals with CKD, who often experience muscle wasting and systemic inflammation; and post-bariatric surgery patients, who face rapid lean mass loss. However, most available evidence relies on surrogate markers of body composition, lacks standardized sarcopenia criteria, and fails to address long-term effects on clinically meaningful endpoints like disability, falls, and quality of life.

To advance this field, future studies should investigate optimal dosing strategies, treatment durations, and the efficacy of combining GLP-1RAs with structured resistance training and nutritional support. The exploration of newer GLP-1RA formulations—including oral semaglutide and dual or triple agonists—may also reveal more favorable therapeutic profiles for muscle preservation.

Mechanistic studies are likewise needed to better understand how GLP-1R activation influences pathways involved in muscle anabolism, mitochondrial function, inflammation, and energy metabolism. Understanding these interactions could open the door to precision medicine approaches targeting sarcopenia in specific clinical contexts.

In summary, while GLP-1RAs hold promise as therapeutic agents for the mitigation of sarcopenia, particularly in CKD, their potential remains underexplored. The current lack of trials specifically designed to evaluate muscle outcomes represents a major limitation. Well-designed, multidisciplinary studies are urgently needed to confirm whether GLP-1RAs can be effectively integrated into broader sarcopenia prevention and treatment strategies. Given the rising burden of sarcopenia and its impact on functional independence and healthcare utilization, this line of research could carry significant public health implications.

## Figures and Tables

**Table 1:** Summary of most relevant trials in CKD or CKD-stratified populations.

Study [Ref.]	Population	Intervention	Renal Outcome
REWIND [17]	T2DM CKD subgroup	Dulaglutide 1.5 mg weekly	15% reduction in composite renal outcome and macroalbuminuria
LEADER [15]	T2DM CKD subgroup	Liraglutide 1.8 mg daily	22% reduction in renal composite and macroalbuminuria
SUSTAIN-6 [16]	T2DM CKD subgroup	Semaglutide 0.5–1.0 mg weekly	36% reduction in renal events including new macroalbuminuria
AMPLITUDE-O [18]	T2DM CKD subgroup	Efpeglenatide weekly	32% reduction in composite renal outcomes
FLOW [19]	T2DM with CKD (eGFR 25–75 mL/min/1.73 m^2^)	Semaglutide 1 mg weekly	24% reduction in hard kidney events and slower eGFR decline
AWARD-7 [72]	T2DM with CKD (eGFR 15–60 mL/min/1.73 m^2^)	Dulaglutide 0.75–1.5 mg weekly vs. insulin	Slower eGFR decline vs. insulin

T2DM: type 2 diabetes mellitus; CKD: chronic kidney disease; eGFR: estimated glomerular filtration rate.

**Table 2:** Summary of major RCTs evaluating GLP-1RAs and muscle-related outcomes in T2DM and obesity.

Study [Ref.]	Population	Intervention	Muscle-Related Findings
SUSTAIN 8 [27]	T2DM	Semaglutide vs. canagliflozin	Preferential fat reduction, increased relative LM
STEP 1 (post hoc DXA study) [26]	Obesity without diabetes	Semaglutide 2.4 mg	Reduction in LM (9.7%) but increased LM/weight (3.0%)
SURMOUNT-1 (DXA substudy) [74]	Obese individuals	Tirzepatide vs. placebo	High fat mass loss (33.9%) but moderate LM reduction (10.9%)
SURPASS-3 and -5 (DXA substudies) [75,76]	T2DM	Tirzepatide	Preservation of LM, fat loss dominance
SURPASS-3 MRI (post hoc) [77,78]	T2DM	Tirzepatide vs. insulin	Improved muscle quality with reductions in fat infiltration, preserved muscle volume
LEADER/SUSTAIN-6 [15,16]	T2DM + high CV risk	Liraglutide/semaglutide	Potential indirect muscle preservation via anti-inflammatory, endothelial protection
AWARD-11 [79]	T2DM	High-dose dulaglutide	Body weight loss with LM preservation
AMPLITUDE-O [18]	High-risk T2DM	Efpeglenatide	Potential indirect muscle benefits derived from anti-inflammatory, renal, and metabolic effects

T2DM: type 2 diabetes mellitus; LM: lean mass; DXA: dual-energy X-ray absorptiometry; MRI: magnetic resonance imaging; CV: cardiovascular.

**Table 3:** Mechanistic actions of GLP-1 receptor agonists relevant to muscle homeostasis.

Mechanism	Description	Relevance to Muscle in CKD
**Insulinotropic**	Enhances insulin secretion and sensitivity, promoting PI3K/Akt/mTOR activation.	Improves anabolic signaling, supports protein synthesis, reduces muscle proteolysis.
**Glycemic Control**	Lowers glucose levels and insulin resistance, reduces glucotoxicity and AGE accumulation.	Restores anabolic pathways, reduces catabolic signaling, oxidative damage, and improves energy metabolism in muscle.
**Anti-Inflammatory**	Inhibits NF-κB, NLRP3 inflammasome, and pro-inflammatory cytokine production.	Reduces inflammation-induced muscle atrophy and catabolic signaling, improves muscle regeneration.
**Antioxidant**	Enhances antioxidant enzymes, reduces ROS via NADPH oxidase inhibition and AMPK activation.	Preserves mitochondrial integrity and muscle fiber structure; limits oxidative stress.
**Mitochondrial** **Biogenesis**	Upregulates PGC-1α via AMPK and SIRT1, enhancing mitochondrial function and ATP production.	Improves muscle endurance and energy metabolism and reduces fatigue and mitochondrial dysfunction.
**Autophagy and Apoptosis Modulation**	Restores autophagic flux and suppresses ER stress-mediated apoptosis through AMPK-mTOR signaling.	Prevents excessive proteolysis and preserves myocyte viability.
**NMJ Support**	Promotes neuronal survival and synaptic function via neuroprotective pathways (cAMP/PKA/CREB, PI3K/Akt).	May preserve NMJ integrity and motor function, indirectly supporting muscle performance in neuromuscular decline.
**Vascular Protection and Perfusion**	Enhances endothelial function, NO production, and capillary recruitment via eNOS, VEGF.	Improves muscle oxygenation, nutrient delivery, and waste removal.

AGE: advanced glycation end products; NF-κB: nuclear factor kappa-light-chain-enhancer of activated B cells; NLRP3: NOD-like receptor family pyrin domain containing 3; ROS: reactive oxygen species; NADPH: nicotinamide adenine dinucleotide phosphate; AMPK: AMP-activated protein kinase; PGC-1α: peroxisome proliferator-activated receptor gamma coactivator 1-alpha; SIRT1: sirtuin 1; ATP: adenosine triphosphate; NMJ: neuromuscular junction; cAMP: cyclic adenosine monophosphate; PKA: protein kinase A; CREB: cAMP response element-binding protein; PI3K: phosphoinositide 3-kinase; NO: nitric oxide; ENOS: endothelial nitric oxide synthase; VEGF: vascular endothelial growth factor.

## Data Availability

Not applicable.
